# The association between anxiety symptoms and sleep quality in people living with HIV/AIDS: the mediating effect of social support, psychological resilience and coping strategies

**DOI:** 10.3389/fpsyt.2026.1840232

**Published:** 2026-09-04

**Authors:** Dan Liu, Xiaoting Chen, Maomao Xi, Lei Shi, Shun Gong, Mengyuan Wu, Dandan Xu, Xiaodong Huang

**Affiliations:** 1 Department of Public Health, Wuhan Third Hospital, Wuhan, Hubei, China; 2 Institute of Burn Research, Wuhan Third Hospital, Wuhan, Hubei, China; 3 Department of Infectious Diseases, Wuhan Third Hospital, Wuhan, Hubei, China; 4 Department of HIV/AIDS Prevention and Control, Wuhan Hongshan Center for Disease Control and Prevention, Wuhan, Hubei, China; 5 School of Public Health, Tongji Medical College, Huazhong University of Science and Technology, Wuhan, Hubei, China; 6 Office of the Hospital Party Committee, Wuhan Third Hospital, Wuhan, Hubei, China

**Keywords:** anxiety, mediating effect, PLWHA, psychological resilience, sleep quality, social support

## Abstract

**Objective:**

This cross-sectional observational study aimed to investigate the relationship between anxiety symptoms and sleep quality among people living with HIV/AIDS (PLWHA), and to examine the parallel mediating roles of social support, psychological resilience, and positive coping style in this association.

**Methods:**

From October 2024 to February 2025, 241 PLWHA were recruited via convenience sampling. Participants completed standardized measures including the Athens Insomnia Scale, Generalized Anxiety Disorder Scale, and scales for depression, perceived social support, coping style, and psychological resilience. All basic demographic statistical analysis, correlation analysis and descriptive statistics were performed using SPSS 23.0. The parallel mediation analysis was performed using R software (version 4.4.2).

**Results:**

The insomnia and non-insomnia groups differed significantly by age, educational level, employment status, and CD4+ T cell count (*p* < 0.05). Sleep quality scores were negatively correlated with social support scores (r=-0.299), positive coping scores (r=-0.313), and resilience scores (r=-0.411),and positively correlated with depressive symptom scores (r=0.693) and anxiety symptom scores (r=0.631), all p-values < 0.001. Depressive symptom scores were positively correlated with anxiety symptom scores (r=0.848) and negatively correlated with social support scores (r=-0.322), positive coping scores (r=-0.308), and resilience assessment scores (r=-0.393), all p-values < 0.001. Social support (effect size=0.037,95% CI: 0.010-0.080), positive coping style (effect size=0.050,95%CI:0.018-00.090), and psychological resilience (effect size=0.069,95%CI:0.035-0.110) partially mediated the relationship between anxiety symptoms and sleep quality, accounting for 7.2%,9.6%, and 13.3% of the total effect respectively.

**Conclusion:**

Social support, psychological resilience and positive coping style partially mediate the statistical association between anxiety symptoms and sleep quality among PLWHA. These three psychosocial protective factors are promising intervention targets for alleviating the adverse correlates of anxiety symptoms on sleep quality in this population.

## 1 Introduction

Human Immunodeficiency Virus (HIV) infection and Acquired Immune Deficiency Syndrome (AIDS) continues to be a major global public health issue. With the widespread adoption of antiretroviral therapies(ART), mortality rates related to HIV/AIDS have decreased, and the disease has evolved into a chronic condition (1, 2). However, PLWHA endure long-term disease stress, persistent social stigma, interpersonal discrimination, and recurrent treatment-related side effects, resulting in a disproportionately high prevalence of psychological and sleep dysfunction compared with the general population (3, 4). Studies show that emotional disorders such as anxiety and depression occur significantly more frequently among PLWHA than the general population, while sleep disorders have become a critical complication affecting their quality of life (2–4). Existing population-specific studies have confirmed that PLWHA exhibit a significantly higher risk of clinical anxiety and sleep disorders, which severely impair their long-term quality of life, disease self-management ability, and immune function stability (2, 4, 5). For instance, a study involving adolescents found a significant correlation between anxiety and sleep quality, with those experiencing higher anxiety levels being more likely to suffer from sleep disorders (2). Accumulating evidence focused on HIV populations has demonstrated a robust statistical association between anxiety symptoms and poor sleep quality: persistent anxiety acts as a crucial psychological stressor that exacerbates sleep latency, sleep fragmentation, and early awakening, ultimately forming a bidirectional adverse correlational cycle in PLWHA (5, 6). Nevertheless, most existing studies only report simple correlations between negative emotions and sleep outcomes among PLWHA, and the underlying psychological mediating mechanisms remain insufficiently explored. This study was grounded in the stress coping theory, which posits that individuals’ physical and mental adaptation to chronic stress depends not only on external stress stimuli but also on internal psychological resources and proactive coping responses. Under the long-term chronic stress of HIV infection, PLWHA are prone to emotional disorders and sleep deterioration; however, positive psychosocial resources can effectively buffer the adverse effects of stress (7, 8).

Social support, psychological resilience, and coping styles serve as core psychosocial resources for individuals to cope with adversity, demonstrating significant moderating roles in chronic disease management, and with relatively independent functional pathways. Specifically, external social support provides emotional comfort and practical assistance to alleviate perceived stress, internal psychological resilience maintains individual emotional stability under persistent disease pressure, and positive coping styles help individuals actively regulate negative emotional responses (5–8). Although these protective factors have been separately verified to correlate with mental health and sleep status in PLWHA populations, few studies have incorporated all three variables into a unified analytical framework based on stress coping theory to systematically compare their independent indirect associations between anxiety and sleep quality.

Therefore, this cross-sectional study adopted a parallel mediation model based on stress coping theory to explore the association between anxiety symptoms and sleep quality and to compare the independent mediating effects of three psychosocial protective factors. The findings provide empirical evidence for targeted psychological intervention strategies to improve sleep health among PLWHA. The parallel model was specifically selected because these three psychosocial factors represent relatively independent stress-buffering resources with distinct functional pathways. Integrating them into a single parallel framework enabled simultaneous estimation and comparative analysis of their indirect associations while controlling for mutual confounding effects, which provided a more systematic and refined understanding of the psychological mechanisms affecting sleep quality among this population. The findings provide novel empirical evidence and theoretical support for targeted psychological intervention research to improve sleep health among PLWHA.

## 2 Materials and methods

### 2.1 Study design and participants

This study adopted a convenience sampling method to recruit participants. From October 2024 to February 2025, a total of 241 PLWHA were enrolled from among 405 consecutive attendees visiting the HIV outpatient department of a tertiary hospital and a Center for Disease Control and Prevention in Wuhan, China, after strict screening based on unified inclusion and exclusion criteria. Inclusion criteria were as follows: (1) Aged 18 years or older; (2) Confirmed HIV-positive diagnosis; (3) Intact cognitive function, full understanding of the study content, and voluntary participation with willingness to complete the questionnaire independently. Exclusion criteria were as follows: (1) Presence of severe cognitive impairment or disturbance of consciousness; (2) Unwillingness to cooperate with questionnaire completion due to personal reasons.

### 2.2 Data collection

All questionnaires were completed throughself-administration with researcher assistance. Before formal investigation, all participating researchers received unified standardized professional training regarding study purpose, implementation procedure, data confidentiality principle, and standardized questionnaire guidance. During data collection, trained researchers fully explained the research purpose, anonymous and confidential data principles, and relevant filling requirements to each eligible participant. All participants independently finished the online questionnaire via the Wenjuanxing platform following unified standardized operating procedures under one-on-one guidance and supervision of researchers. For participants with low educational level or confusion about item understanding, researchers provided standardized and objective explanations without inducing participants’ answers. After questionnaire submission, researchers immediately checked the completeness and validity of the responses. Meanwhile, the collected questionnaire data were cross-verified with the participants’ clinical medical record information to ensure the accuracy and authenticity of the research data. The general questionnaire included demographic characteristics, such as gender, age, educational, Body Mass Index (BMI), marriage, occupation, and CD4+ T cell count.

The Athens Insomnia Scale (AIS-8) was used to assess the severity of insomnia. It was developed by Professor Dan Sedmark, Vice Dean of the School of Medicine at Ohio State University, USA (9). The scale contains 8 items, the first five items assess nighttime sleep status, while the last three items evaluate daytime functioning. Each item is scored from 0 to 3 points, with a total scale score ranging from 0 to 24 points. Higher total AIS-8 scores indicate poorer sleep quality, and a total score exceeding 6 points is indicative of sleep disorders, and the Cronbach’s α coefficient is 0.90.

The Patient Health Questionnaire Depression Scale (9-item Patient Health Questionnaire, PHQ-9) was used to measure patients’ depression levels. It was developed by K. Kroenke et al. in 2001 (10), which contains 9 items, with a score of 0–3 for each item, and the total score ranges from 0 to 27. Consistent with widely recognized clinical cutoff standards, a total PHQ-9 score > 10 was defined as the presence of clinically significant depressive symptoms in this study,and the Cronbach’s α coefficient is 0.87.

The Generalized Anxiety Disorder Scale (GAD-7)was used to measure patients’anxiety levels. The scale was developed by Spitzer et al. in 2006 (11), which contains 7 items, with a score of 0–3 for each item, and the total score ranges from 0 to 21. Referencing standard clinical screening criteria, a total GAD-7 score > 10 was adopted as the cutoff point to identify individuals with clinically significant anxiety symptoms. and the Cronbach’s α coefficient is 0.96.

The Perceived Social Support Scale (PSSS-12) was used to measure the level of social support of patients. The scale was developed by Zimet et al. in 1988 (12), which contains 4 questions on the family subscale, 4 questions on the friends subscale, and 4 questions on the other subscales, for a total of 12 questions. Each topic is scored 1–7, and the total score ranges from 12 to 84. In this study, when the total score is more than 61 or more, it indicates a high support level, and the Cronbach’s α coefficient is 0.93.

The Simplified Coping Style Questionnaire (SCSQ) is used to assess individual coping styles in the face of life emergencies. The scale was developed by Jie Yaning (13). The scale contains 20 items, and is divided into two dimensions, the positive coping and negative coping. The positive coping dimension consists of items 1-12, and the negative coping dimension consists of items 13-20, and the Cronbach’s α coefficient of 0.90.

The 10-item Connor-Davidson Resilience Scale (CDRISC-10) was developed by Campbell-Sills & Stein (2007), which contains 10 items. It covers important dimensions of resilience, such as tenacity, self-reliance and optimism. The total score ranges from 0 to 40, with higher scores indicating greater psychological resilience (13). The Cronbach’s α coefficient is 0.90.

### 2.3 Statistical analysis

Categorical variables were presented as percentages. The chi-square (χ²) test was used for intergroup comparisons. Pearson correlation analysis was performed to evaluate the relationships between social support, psychological resilience, positive coping strategies, and sleep quality. All basic demographic statistical analysis, correlation analysis and descriptive statistics were performed using SPSS 23.0. The parallel mediation analysis was performed using R software (version 4.4.2). The mediation model was established using the corresponding mediation analysis package, and bootstrap resampling with 5000 iterations was applied to verify the mediation effect. The significance level was set at α = 0.05 for all statistical tests.

### 2.4 Ethics

This study complied with the World Medical Association’s Declaration of Helsinki’s Ethical Principles and Good Clinical Practice for medical research in humans and all applicable regulations. The clinical research protocol and informed consent form were both approved by the Ethics Committee of the Wuhan third Hospital. The subjects recruited for the clinical trial voluntarily signed the informed consent form. (KY2022-060).

## 3 Results

### 3.1 Common method bias test

All items were subjected to Harman’s single-factor test. Nine common factors with eigenvalues>1 were identified through non-rotated exploratory factor analysis. The first common factor explained 32.18% of the variance (<40%), indicating no significant common method bias.

### 3.2 Study participant enrollment, characteristics and scale scores for each dimension

The questionnaire was distributed to 245 PLWHA, and 241 valid questionnaires were collected, with a recovery rate of 98.37%. **Table 1** presents the demographic characteristics and dimensional scale scores of the PLWHA. Among the 241 patients, the majority were male, ages were primarily between 18–50 years old, and they were predominantly unmarried. Their education was mainly college-level or higher. Some PLWHA also had other chronic diseases. The results showed significant differences *(p* < 0.05) between PLWHA with and without sleep disorders in terms of age, educational level, employment status, and CD4+ T cell count.

**Table 1:** General information and differences in PLWHA.

Characteristics	Total (n=241)	Non sleep disorders (n=133)	Sleep disorders (n=108)	*p* value
Sex, n(%)				0.234
Male	229 (95.0%)	124 (93.2%)	105 (97.2%)	
Female	12 (5.0%)	9 (6.8%)	3 (2.8%)	
Age (years), n(%)				0.021
<50	209 (86.7%)	109 (82.0%)	100 (92.6%)	
≥50	32 (13.3%)	24 (18.0%)	8 (7.4%)	
Body Mass Index (BMI), n(%)				0.121
<18.5	25 (10.4%)	11 (8.3%)	14 (13.0%)	
18.5-23.9	168 (69.6%)	89 (66.9%)	79 (73.1%)	
24-27.9	38 (15.8%)	25 (18.8%)	13 (12.0%)	
≥28	10 (4.2%)	8 (6.0%)	2 (1.9%)	
Marital status, n(%)				0.853
Others	207 (85.9%)	115 (86.5%)	92 (85.2%)	
Married	34 (14.1%)	18 (13.5%)	16 (14.8%)	
Education level, n(%)				0.022
Junior high school and below	18 (7.5%)	11 (8.3%)	7 (6.5%)	
High School/Vocational School	28 (11.6%)	22 (16.5%)	6 (5.6%)	
College degree or higher	195 (80.9%)	100 (75.2%)	95 (88.0%)	
Fixed employment status, n(%)				0.001
Yes	133 (55.2%)	86 (64.7%)	47 (43.5%)	
No	108 (44.8%)	47 (35.3%)	61 (56.5%)	
Viral load (copies/mL), n(%)				0.429
≤50	226 (93.8%)	123 (92.5%)	103 (95.4%)	
>50	15 (6.2%)	10 (7.5%)	5 (4.6%)	
CD4+ T cell count (cells/μl), n(%)				0.036
≤199	11 (4.6%)	6 (4.5%)	5 (4.6%)	
200-349	32 (13.3%)	11 (8.3%)	21 (19.4%)	
350-499	59 (24.5%)	30 (22.6%)	29 (26.9%)	
≥500	139 (57.6%)	86 (64.7%)	53 (49.1%)	
Other chronic comorbidities, n(%)				0.979
Yes	40 (16.6%)	22 (16.5%)	18 (16.7%)	
No	201 (83.4%)	111 (83.5%)	90 (83.3%)	
PHQ-9 scores	6.0 (3.0, 9.0)	4.0 (0.0, 6.0)	9.0 (6.0, 12.0)	<0.001
GAD-7 scores	4.0 (1.0, 7.0)	2.0 (0.0, 5.0)	7.0 (4.0, 8.0)	<0.001
PSSS-12 scores	53.0 (48.0, 62.0)	57.0 (48.0, 65.0)	48.0 (44.0, 57.0)	<0.001
CD-RISC-10 scores	27.0 (21.0, 31.0)	30.0 (25.0,33.0)	23.5 (20.0, 29.0)	<0.001
SCSQ scores				
positive coping scores	34.0 (28.0, 39.0)	35.0 (30.0, 39.0)	31.0 (26.0, 37.3)	0.007
negative coping scores	18.0 (16.0, 21.0)	17.0 (15.0, 19.0)	19.5 (16.00, 22.3)	<0.001

The scale scores did not follow a normal distribution. Continuous data were presented as median (lower quartile, upper quartile). The Mann-Whitney U test was used to compare differences between the sleep disorder group and the non-sleep disorder group.

### 3.3 Analysis of the correlation between sleep quality, anxiety symptoms, depression symptoms, social support, coping style and psychological resilience

The correlation analysis results are shown in **Table 2**. The Pearson correlation analysis shows a negative correlation between AIS-8 scores and PSSS-12 scores (*r* = -0.299, *p* < 0.001), positive coping style scores (*r* = -0.313, *p* < 0.001), and psychological resilience scores (*r* = -0.411, *p* < 0.001), and show a positive correlation with PHQ-9 scores (*r* = 0.693, *p* < 0.001) and GAD-7 scores (*r* = 0.631,*p* < 0.001). PHQ-9 scores and GAD-7 scores show a positive correlation (*r* = 0.848, *p* < 0.001), and show a negative correlation between PSSS-12 scores (*r* = -0.322, *p* < 0.001), positive coping style scores (*r* = -0.308, *p* < 0.001), and resilience scores (*r* = -0.393, *p* < 0.001). Higher PHQ-9 scores and GAD-7 scores were positively correlated with elevated AIS-8 scores, indicating that increased negative emotional status was associated with poorer sleep quality. In contrast, Higher PSSS-12 scores, positive coping style scoresand psychological resilience scores were negatively correlated with AIS-8 scores, suggesting that better psychological resources contributed to improved sleep quality.

**Table 2:** Correlation analysis of variables among PLWHA.

Variable	AIS-8 scores	PHQ-9 scores	GAD-7 scores	PSSS-12 scores	Positive coping scores	CDRISC-10 scores
AIS-8 scores	1.00					
PHQ-9 scores	0.69 (0.62 to 0.75)	1.00				
GAD-7 scores	0.63 (0.55 to 0.70)	0.85 (0.81 to 0.88)	1.00			
PSSS-12 scores	-0.30 (-0.41 to -0.18)	-0.32 (-0.43 to -0.20)	-0.30 (-0.41 to -0.18)	1.00		
Positive coping scores	-0.31 (-0.42 to -0.19)	-0.31 (-0.42 to -0.19)	-0.29 (-0.40 to -0.17)	0.42 (0.31 to 0.52)	1.00	
CDRISC-10 scores	-0.41 (-0.51 to -0.30)	-0.39 (-0.49 to -0.28)	-0.35 (-0.45 to -0.23)	0.46 (0.36 to 0.56)	0.66 (0.58 to 0.72)	1.00

Correlations were presented as correlation coefficients (r) with 95% confidence intervals.

### 3.4 Testing the mediation effect model

**Figure 1:**
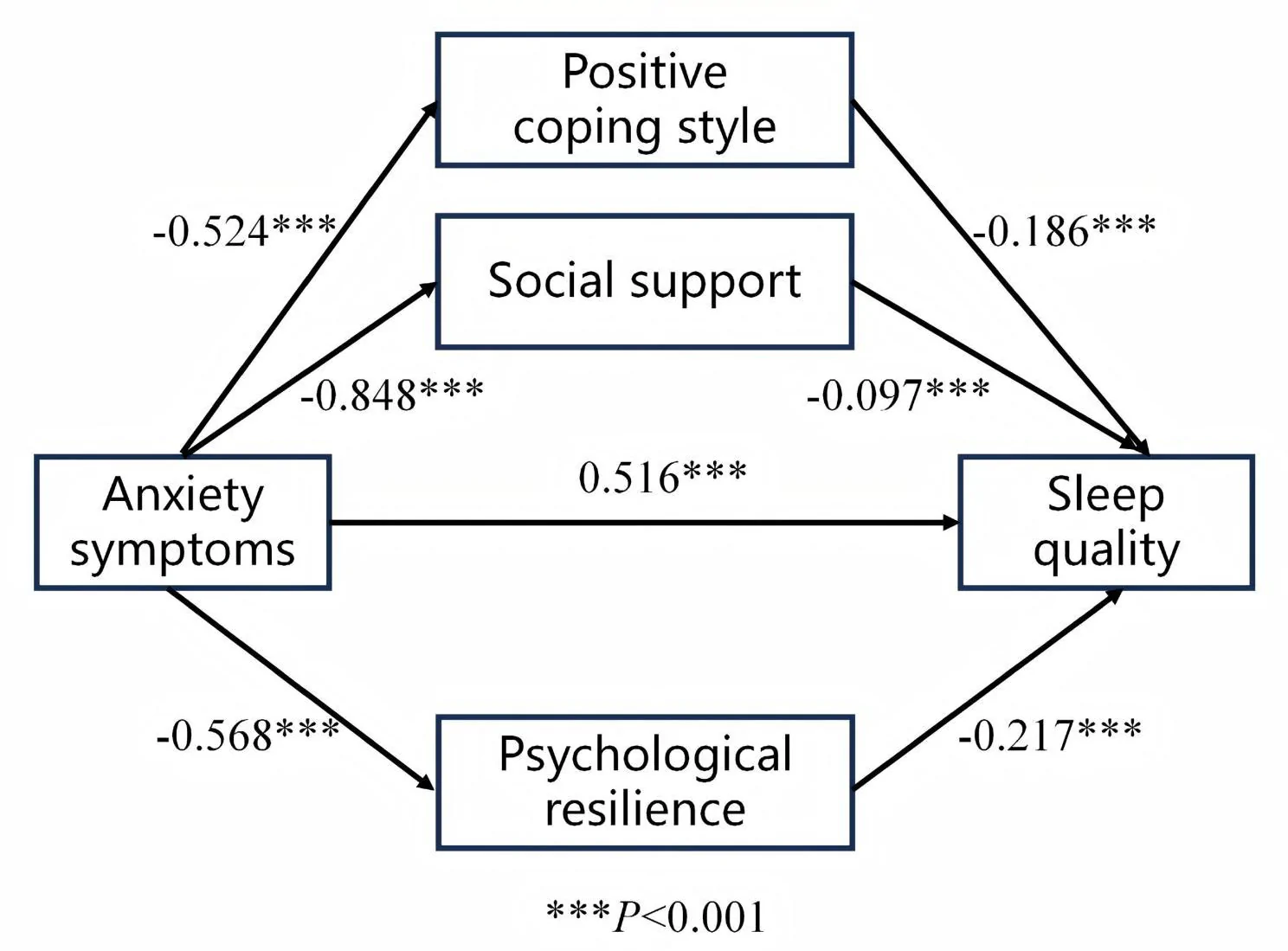
Parallel mediating effect model of social support, positive coping style and psychological resilience between anxiety symptoms and sleep quality.

Three independent simple mediation models were constructed using the mediation package in R. Anxiety as the independent variable, sleep quality as the dependent variable, and social support, positive coping style, and psychological resilience as the mediators. Referring to previous studies, age, educational level, and employment status were included as covariates. Bootstrap resampling with 5000 iterations was performed to test the mediating effects of social support, psychological resilience, and positive coping style. The statistical significance level was set at α = 0.05.The mediation model is depicted in **Figure 1**. The results showed that anxiety scores were positively associated with the sleep quality scores (*β* = 0.516, *p* < 0.001),indicating that higher anxiety levels are associated with worse sleep quality among PLWHA. Besides, anxiety scores were negatively associated with the social support scores (*β* = -0.848, *p* < 0.001), positive coping style scores (*β* = -0.524, *p* < 0.001) and psychological resilience scores (*β* = -0.568, *p* < 0.001). The social support scores (*β* = -0.097, *p* < 0.001), positive coping style scores (*β* = -0.186, *p* < 0.001), and psychological resilience scores (*β* = -0.217, *p* < 0.001) were negatively associated with the sleep quality scores, indicating that higher levels of social support, positive coping strategies, and psychological resilience contribute to improved sleep quality. Detailed path coefficient results are presented in **Table 3**.

**Table 3:** Correlation analysis among variables.

Paths	β	95%CI	t	*p*
Anxiety symptoms→Social Support→Sleep quality				
Anxiety symptoms→Social Support	-0.848	-1.186 to -0.510	-4.940	<0.001
Social Support→Sleep quality	-0.097	-0.133 to -0.060	-5.219	<0.001
Anxiety symptoms→Sleep quality (direct)	0.516	0.431 to 0.600	12.074	<0.001
Anxiety symptoms→Positive Coping style→Sleep quality				
Anxiety symptoms→Positive Coping style	-0.524	-0.722 to -0.325	-5.197	<0.001
Positive Coping style→Sleep quality	-0.186	-0.247 to -0.125	-6.019	<0.001
Anxiety symptoms→Sleep quality (direct)	0.516	0.431 to 0.600	12.074	<0.001
Anxiety symptoms→Psychological resilience → Sleep quality				
Anxiety symptoms→Psychological resilience	-0.568	-0.763 to -0.373	-5.738	<0.001
Psychological resilience→Sleep quality	-0.217	-0.277 to -0.158	-7.171	<0.001
Anxiety symptoms→Sleep quality (direct)	0.516	0.431 to 0.600	12.074	<0.001

As shown in **Table 4**, the mediating effects were further tested using the bootstrap method, where the mediating effects model was tested using repeated random sampling 5000 times in the raw data. The results showed that the path of anxiety affecting sleep quality is significant, and social support, positive coping style, and psychological resilience played partial mediating roles between anxiety and sleep quality. Social support (effect size=0.037, 95%*CI*: 0.010, 0.080), positive coping style (effect size=0.050, 95%*CI*: 0.018, 0.090), and psychological resilience (effect size=0.069, 95%*CI*: 0.035, 0.110) partially mediate the impact of anxiety between sleep quality, with an effect contribution of 7.2%, 9.6%, and 13.3%.

**Table 4:** Decomposition of mediating effect, total effect and direct effect.

Variable	Path	Estimate (95% CI)	*p* value	Proportion(%)
Direct effect	Anxiety symptoms→Sleep quality	0.478 (0.361-0.580)	<0.001	92.8
Indirect effect	Anxiety symptoms→Social support→Sleep quality	0.037 (0.010-0.080)	0.005	7.2
Total effect		0.516 (0.416-0.610)	<0.001	100.0
Direct effect	Anxiety symptoms→ Sleep quality	0.466 (0.361-0.560)	<0.001	90.4
Indirect effect	Anxiety symptoms→Positive coping style→Sleep quality	0.050 (0.018-0.090)	0.002	9.6
Total effect		0.516 (0.416-0.610)	<0.001	100.0
Direct effect	Anxiety symptoms→Sleep quality	0.447 (0.341-0.550)	<0.001	86.7
Indirect effect	Anxiety symptoms→Psychological resilience→Sleep quality	0.069 (0.035-0.110)	<0.001	13.3
Total effect		0.516 (0.416-0.610)	<0.001	100.0

## 4 Discussion

### 4.1 Analysis of variance between the sleep-disordered group and non-sleep-disordered group of PLWHA

This study compared sleep-related and clinical characteristics between PLWHA with and without sleep disorders, and identified significant intergroup differences in age distribution, CD4+ T lymphocyte counts, education level and employment status. A higher proportion of PLWHA aged younger than 50 years were observed in the sleep disorder group. Younger PLWHA may experience greater sleep disturbances, which could be related to psychosocial stress, challenges adapting to chronic disease, and unhealthy lifestyle factors. Younger PLWHA frequently confront career barriers, financial instability, and social stigma, which may aggravate anxiety and depressive symptoms and further impair sleep quality (2, 8). Additionally, disease adaptation challenges were particularly prominent among younger groups. Newly diagnosed PLWHA need to adapt to life changes from long-term treatment, and this adaptation process may further induce mood fluctuations and sleep disturbances (6, 8, 14). Lifestyle factors also cannot be ignored. As documented in previous studies, adolescents are prone to behavioral patterns such as nighttime electronic device use and irregular sleep patterns, which can disrupt normal sleep rhythms and further impair sleep quality (15). This study further found that PLWHA in the non-sleep disorder group exhibited higher CD4+ T lymphocyte counts than those in the sleep disorder group. This result is inconsistent with a previous study by Korkmaz et al., which reported greater CD4+ T lymphocyte reconstitution among HIV-infected individuals with poor sleep quality. Such discrepancies may be attributed to differences in immune reconstitution stages and baseline inflammatory levels across study populations. The lower CD4+ T lymphocyte counts observed in the sleep disorder group may be explained by the inhibitory effects of insufficient or disturbed sleep on immune function via neuroendocrine pathways (16, 17). The proportion of college-educated PLWHA with sleep disorders was significantly higher than in non-disorder groups, maybe related to heightened health sensitivity or greater work pressure among highly educated populations. The proportion of PLWHA with sleep disorders who lack stable employment was significantly higher than those without sleep disorders, which may be linked to anxiety caused by economic stress, insufficient social support, or unemployment. In addition, the adherence to ART treatment among unemployed individuals may be reduced, and medication lapses leading to viral fluctuations can affect the sleep center through neuroinflammatory pathways (18, 19).

### 4.2 Anxiety symptoms are positively associated with poor sleep quality among PLWHA

This study indicates that anxiety scores in PLWHA positively correlate with sleep quality (r=0.631, P<0.001), with higher anxiety levels correlating to poorer sleep quality. This may be related to persistent stress such as disease stigma, treatment burdens, and social relationship changes (1, 3, 5). Anxiety can disrupt the cortisol secretion rhythm, and directly inhibiting slow-wave activity essential for deep sleep, thereby reducing sleep quality (20). Notably, anxiety and sleep disorders can exhibit bidirectional associations, and fragmented sleep can suppress the prefrontal cortex function, impairing the emotional regulation and exacerbating the anxiety symptoms (21).

### 4.3 Social support, positive coping style, and psychological resilience played a mediating role between anxiety symptoms and sleep quality in PLWHA

The study indicates that social support, positive coping, and psychological resilience serve as three mediating variables influencing the sleep quality. Anxiety symptoms affect sleep quality in PLWHA by reducing social support, diminishing positive coping styles, and weakening psychological resilience. Research indicates that anxiety can impair patients ‘ability to seek social support proactively, leading to a shrinking support network and worsening sleep quality (effect size=0.037). Previous studies have shown that social support can directly influence the level of anxiety as well as the level of depression in PLWHA. Furthermore, the support of family and friends is extraordinarily important for the PLWHA. It can even be said that it can influence all aspects of PLWHA, such as how they deal with stigma, whether they take medication as required, and whether they engage in suicidal behaviour (7, 22). Therefore, we suggest that a multi-level and institutionalized social support system can be built, such as implementing family members’ psychological education to enhance HIV awareness, reduce internal family stress, improve support quality, and elevate social support levels.

This study indicates that positive coping style played a mediating role in the relationship between anxiety and sleep (effect size=0.050). Anxiety can weaken the PLWHA’ capacity to adopt positive coping styles, directly impairing sleep regulation abilities. The possible explanation for these findings is that positive coping style is significantly associated with optimism, and optimism may reduce anxiety symptoms because it encourages people to adopt positive ways of coping in the face of stress (23). Therefore, targeted interventions focusing on cognitive reappraisal training and positive coping strategy guidance may help alleviate adverse psychological problems and improve sleep quality among this population (23).

This study indicates that psychological resilience played a mediating role in the relationship between anxiety and sleep, the psychological resilience being the strongest mediator in this study (effect size=0.069). This indicates that psychological resilience serves as a core protective factor mitigating the negative impact of anxiety on sleep. Relevant research suggests that strong psychological resilience can reduce the occurrence of anxiety or depression (23). This finding reveals that, in addition to targeted intervention on anxiety symptoms, enhancing individuals’ inner psychological strength—including improving adversity adaptability, maintaining optimism, and strengthening emotional regulation abilities—can more effectively break the vicious cycle of “anxiety-sleep deterioration” among PLWHA. Consequently, it provides a critical entry point for developing targeted psychological intervention programs. Future support strategies should focus on empowering patients and enhancing their psychological flexibility to ultimately improve overall quality of life and health outcomes (6, 24).

### 4.4 Strengths and limitations

This study innovatively identifies the mediating roles of social support, positive coping style, and psychological resilience, and explores their potential associations with sleep quality among PLWHA. These findings deepen the current understanding of the psychological factors influencing sleep quality among this population and provide empirical evidence for targeted psychological intervention strategies. This study has several limitations. First, the cross-sectional design limits the ability to infer causality, and only correlational associations can be concluded. Second, the sample was recruited from a single city in China with a limited sample size, which may restrict the generalizability of the findings. Third, all variables were self-reported, which may introduce potential reporting bias. Finally, several lifestyle and clinical indicators (e.g., electronic device use, ART adherence, viral load) were not included in the current analysis. Future longitudinal and interventional studies are warranted to validate the causal pathways and optimize targeted psychological interventions.

In conclusion, this cross-sectional study systematically explored the association between anxiety symptoms and sleep quality among PLWHA, and verified the partial parallel mediating effects of social support, positive coping style, and psychological resilience. Among the three psychosocial protective factors, psychological resilience exhibited the most prominent protective correlational effect. The study findings clarify the potential psychological mechanism linking anxiety symptoms and sleep disturbances in this population, providing reliable observational evidence for sleep health management of PLWHA. Although these psychosocial factors cannot be easily modified in a single intervention, they serve as valuable and targeted psychosocial intervention directions for future stratified psychological intervention research. Given the limitations of the cross-sectional design, which cannot confirm causal relationships, further longitudinal follow-up studies and targeted intervention trials are essential to verify the exact causal pathways. Exploring optimized psychological intervention strategies based on the three mediating factors will help alleviate sleep problems induced by anxiety symptoms, and ultimately improve the overall mental health and quality of life among PLWHA.

## Data Availability

The original contributions presented in the study are included in the article/supplementary material. Further inquiries can be directed to the corresponding author.
